# Bacteria on Medical Professionals' White Coats in a University Hospital

**DOI:** 10.1155/2020/5957284

**Published:** 2020-10-29

**Authors:** Shyam Kumar Mishra, Sabindra Maharjan, Santosh Kumar Yadav, Niranjan Prasad Sah, Sangita Sharma, Keshab Parajuli, Jeevan Bahadur Sherchand

**Affiliations:** ^1^Department of Microbiology, Institute of Medicine, Tribhuvan University, Kathmandu, Nepal; ^2^School of Optometry and Vision Science, University of New South Wales Sydney, Sydney, Australia; ^3^Department of Microbiology, Rajarshi Janak University, Janakpurdham, Nepal

## Abstract

The transient contamination of medical professional's attires including white coats is one of the major vehicles for the horizontal transmission of microorganisms in the hospital environment. This study was carried out to determine the degree of contamination by bacterial agents on the white coats in a tertiary care hospital in Nepal. Sterilized uniforms with fabric patches of 10 cm × 15 cm size attached to the right and left pockets were distributed to 12 nurses of six different wards of a teaching hospital at the beginning of their work shift. Worn coats were collected at the end of the shifts and the patches were subjected for total bacterial count and identification of selected bacterial pathogens, as prioritized by the World Health Organization (WHO). Fifty percent of the sampled swatches were found to be contaminated by pathogenic bacteria. The average colony growth per square inch of the patch was 524 and 857 during first and second workdays, respectively, indicating an increase of 63.6% in colony counts. The pathogens detected on patches were *Staphylococcus aureus*, *Escherichia coli*, *Pseudomonas aeruginosa*, and *Acinetobacter* sp. Additional bacteria identified included *Bacillus* sp., *Micrococcus* sp., and coagulase-negative *staphylococci* (CoNS). The nurses working in the maternity department had their white coats highly contaminated with bacteria. On the other hand, the least bacterial contamination was recorded from the nurses of the surgery ward. One *S. aureus* isolate from the maternity ward was resistant to methicillin. This study showed that pathogens belonging to the WHO list of critical priority and high priority have been isolated from white coats of nurses, thus posing the risk of transmission to patients. White coats must be worn, maintained, and washed properly to reduce bacterial contamination load and to prevent cross-contamination of potential superbugs. The practice of wearing white coats outside the healthcare zone should be strictly discouraged.

## 1. Introduction

Healthcare-associated infections (HAIs) are the most frequent adverse events in healthcare delivery. It has been estimated that globally hundreds of millions of patients are affected by HAIs each year leading to unnecessary mortality and economic burden to healthcare systems, patients, and their families. Around 7% and 10% of hospitalized patients at any given time acquire at least one HAI in developed and developing countries, respectively [1]. The impact of HAIs is more severe in resource-limited settings where the rate of infection is estimated to range from 25% to 40% [2, 3]. Patients shed infectious microorganisms into the healthcare environment, and healthcare workers acquire these organisms, thereby transmitting the microorganisms further [4]. With the increasing prevalence of multidrug-resistant (MDR) bacteria in hospital settings, investigation of the role of environmental factors including medical professionals' attire in the spread of infection is important [5].

Medical professionals' white coat is associated with humanism and standard of professionalism [6]. It is a symbol of hope and healing for medical professionals. At the same time, the bitter irony is white coats are also known to harbor pathogenic bacteria transiently, thus transmitting the microbes in the hospital environment and also predispose to the patients-to-patients transmission of infections [7, 8]. Although studies have not directly linked healthcare workers' apparel to HAIs, different research groups have found that white coats are often colonized with pathogenic bacteria including methicillin-resistant *Staphylococcus aureus* (MRSA) and vancomycin-resistant *enterococci* (VRE) [9, 10]. Among the health workers, nurses are the ones who come in direct contact with the patients during treatment and care. Their uniforms are prone to contamination which increases throughout the shift and contributes to the spread of microorganisms. It has been suggested that nurses' uniform is the missing link in breaking the chain of HAIs [11].

To the best of our knowledge, no studies have reported the bacterial contamination of medical professionals' white coats in healthcare settings in our country. Hence, this study was designed to assess the degree of contamination by bacterial agents on the white coats worn by nurses and the rate by which contamination is increasing by the end of first- and second-day work shifts in a tertiary care hospital of Nepal. The study was also focused to investigate if the white coats are contaminated with any potentially pathogenic bacteria.

## 2. Materials and Methods

### 2.1. Study Design and Study Population

This cross-sectional study was conducted at the Clinical Microbiology Department of Tribhuvan University Teaching Hospital, a 700-bedded hospital located in Kathmandu, Nepal. A total of 12 nurses (two from each of medicine, pediatric, maternity, intensive care unit (ICU), surgery, and postoperative departments) were recruited in this study. Participants were informed of the purpose of the study, and their consent was taken before being enrolled in the study. All the 12 nurses worked for 8 hours on one day shift. Each nurse was asked some questions regarding the maintenance and handling of white coat uses.

### 2.2. Sampling Method

Polyester cotton blend fabrics were purchased from manufacturers of white coats. Fabrics were prepared by boiling with a soap water solution for 30 minutes followed by rinsing with successive hot and cold water and finally dried. Fabric patches of 10 cm × 15 cm sizes were cut from prepared blend fabric. These patches were autoclaved and sealed in sterile plastic bags. The nurses who participated in the study were instructed to launder their coat before the sampling. At the start of the work shift, fabric patches were stitched over the side pockets of the lower left and right sides of the white coat of all nurses. These areas were chosen because microbial contaminations are thought to be greatest there. The participating nurses wore the coat during their 8-hour shift in the ward. After finishing their shift duty, the patch on the right side was removed, kept in a sterile plastic container/Petri dish, and transported to the microbiology laboratory. The white coat was folded and kept securely in the changing room. The following day, the same nursing staff wore the coat during her 8-hour second shift duty. At the end of the shift, the patch from the left side pocket was collected in another well-labelled sterile plastic container and transported to the laboratory [12].

### 2.3. Determination of Bacterial Load on Fabric Patches

When the sampled fabric patches were brought to the laboratory, they were cut out into pieces of a 2 inch × 2 inch portion with sterilized scissors. Then, a piece of 2 inch × 2 inch fabric patch was cut into further several pieces and placed in a 100 ml screw-capped sterile container containing 25 ml of sterile peptone water. To maintain sterility during procedures, gloves were changed and scissors were flamed in between cutting the patches. The fabric pieces in peptone water were vigorously agitated to extract bacteria in the vortex mixture.

Then to determine the viable count of the bacteria, three different volumes of each sample were pipetted on three separate sterile Petri dishes (1000 *µ*l, 100 *µ*L, and 20 *µ*L). About 15 ml of molten nutrient agar medium was added to each plate, the plates were swirled, and allowed to solidify. The plates were incubated for 24 hours at 37°C [13]. After incubation, the average colony count of bacterial isolates was estimated from those different plates. The calculation was done for the bacterial load on the fabric patch and expressed in terms of colony-forming units per square inch (CFU/inch^2^ = total bacterial count in 25 ml peptone water sample/4 inch^2^).

### 2.4. Isolation of Pathogenic Bacteria from Fabric Patches

The sampled fabric swatches were subsequently tested for the presence of any potentially pathogenic bacteria. For this, several pieces of fabric patches were put in a sterile test tube with phosphate-buffered saline for 5 minutes, and then each patch was placed onto a blood agar plate, a MacConkey agar plate, and selective media like mannitol salt agar (for *Staphylococcus aureus*) and cetrimide agar (for *Pseudomonas aeruginosa*) with the exposed side of patches facing the media. The swatches were discarded from the agar plates, and the inoculated plates were dried and incubated for 24 hours at 37°C [12].

### 2.5. Identification of Bacterial Isolates

After incubation, each morphotype of bacterial colonies grown on the media was selected and further processed for identification of bacterial isolates by observation of colony characteristics, Gram's reaction, motility, and different biochemical tests (catalase test, coagulase test, oxidase test, motility test, sugar fermentation test, indole production, citrate utilization, and urea hydrolysis) [14]. The control measures for this study were a sterile fabric patch, solid media plates, and phosphate-buffered saline which were also incubated for 24 hours at 37°C. If there was no growth of microbes on any of the three controls, the probability of contamination before sample collection and during the isolation process was ruled out.

### 2.6. Data Analysis

The data generated during the study were entered and analyzed by Microsoft Excel and manual method and interpreted according to frequency distribution and percentage.

### 2.7. Ethical Statement

The permission for the study was taken from the Department of Clinical Microbiology, Institute of Medicine, Tribhuvan University, Kathmandu, Nepal. Informed consent was obtained from each nurse before enrollment in the study.

## 3. Results

A total of 12 nurses of age between 20 and 37 years participated in this study, which included two from each of the medicine, pediatric, maternity, ICU, surgery, and postoperative departments. From those 12 nurses, 12 fabric patches were sampled after the first-day work shift and an equal number after the second-day work shift. The plate count revealed bacterial contamination of all the fabric patches from the nurses after their first and second work shifts.

The average colony count per square inch of the patch was 524 and 857 during the first and second workdays, respectively. The highest degree of average bacterial contamination was seen on fabrics collected from nurses of the maternity ward after the second-day work shift (1942.5 CFU/inch^2^), and a significantly lower degree of average bacterial contamination was seen on fabric patches collected from the surgery ward after the first-day work shift (103.5 CFU/inch^2^). The degree of bacterial contamination increased on all the sampled patches collected after the second shift as compared to those collected after the first-day work shift. After the second shift, the average increase of bacterial colonies on the fabrics was 63.6%. The increase in bacterial counts after the second shift was higher (by 248.2%) on the patches collected from nurses of ICU and lower (by 18.5%) on the patches from the pediatric ward (Table 1).

Out of fabric patches sampled from 12 nurses, white coats of 50% of nurses were found to be contaminated by pathogenic bacteria. The pathogenic bacteria like *Escherichia coli* and *Pseudomonas aeruginosa* were isolated only during the first-day shift on patches collected from medicine and postoperative wards, respectively. *Staphylococcus aureus* isolate was recovered during the first shift from ICU and during the second shift from the maternity department, while *Acinetobacter* sp. was isolated only from the pediatric department during both first and second shifts. One *S. aureus* isolate from the maternity ward was found to be methicillin-resistant (MRSA). Additional opportunistic pathogens like coagulase-negative staphylococci (CoNS) and nonpathogenic bacteria included *Bacillus* sp. and *Micrococcus* sp., and they were grown from all the patches collected from six wards (Table 2).

While exploring the perception towards handling of white coats, 55.6% of nurses report washing their coats after 3 or fewer days; 33.3% use their coats outside the hospital wards too, and all the nurses possess more than one white coat. Nearly 45% of the nurses were found to have a notion that white coats should be cleaned even if the collar and pocket of the white coat are still clean (Table 3). Ten out of 12 nurses were involved in dealing with more than 10 patients.

## 4. Discussion

White coats have been considered as a symbol of identity, dignity, and credibility to healthcare professionals, and they are subject of hope and healing to patients. However, white coats have been shown to carry highly pathogenic microorganisms too, which can have a role in nosocomial infections [7, 15, 16]. The main offender in the dissemination of potential nosocomial pathogens by the wearer of the white coat is improper handling practices [7, 16].

In many countries, the nurse-to-patient ratio is so low that nurses have excessive workload and hence the handling of gowns and white gowns may not be excellent despite their untiring efforts. In an overcrowded healthcare facility such as government tertiary care hospitals of developing countries, nurses have frequent interactions with sick patients, medical professionals, and visitors, and their white coats have a great chance of contamination by pathogens and transmission across the healthcare settings. In this study, we have assessed the degree of contamination by bacterial agents on the white coats worn by the nurses and the rate by which bacterial contamination is increased by the end of work shifts.

The findings of our study clearly show that white coats used by nurses harbor a high load of bacterial agents and the degree of contamination is reasonably high which agrees with the findings of other studies done by Treakle et al. [4], Wiener-well et al. [5], Munoz-price et al. [17], and Gupta et al. [18]. The high rates of bacterial contamination of coats may be partially explained by the patient's continuous shading of pathogenic microbes in the hospital environment and inadvertent carriage of such bugs by the healthcare workers who are constantly in contact with patients. To reduce bacterial contamination in white coats, strategies like impregnating fabrics with antimicrobial chemicals, e.g., organosilane-based quaternary ammonium, fluoroacrylate copolymer emulsion, silver alloy complex compounds, and chitosan have also been adopted, but with a limited positive outcome [19].

In this study, the average colony growth per square inch of the patch in each ward was found to be 524 after a first-day work shift and 857 after a second-day work shift. Nurses from the maternity department have their white coats highly contaminated, while those from the surgery ward recorded the least rate of contamination. The highest bacterial contamination was seen on white coats of nurses from ICU by Gupta et al. [12]. In our study, bacterial contamination was increased on all collected patches after the second shift, indicating a 63.6% increase than the first-day shift. This increment was highest (by 248.2%) on the patches collected from nurses of ICU which is worrisome. Gupta et al. [12] reported an increase of 90.0% bacterial contamination on nurses' white coats made up of polyester fabric after the second-day shift.

Most of the bacteria isolated from contaminated fabric patches were opportunistic pathogens like CoNS and normal skin flora plus environmental contaminants, e.g., *Bacillus* sp. and *Micrococcus* sp., but potential pathogens were identified as well. In this study, white coats of 50% of nurses were found to be contaminated by pathogens like *S. aureus*, *E. coli*, *P. aeruginosa*, and *Acinetobacter* sp. White coats of nurses from pediatrics, medicine, maternity, ICU, and postoperative wards were found to have been contaminated by pathogenic bacteria. One *S. aureus* isolate from the maternity ward was methicillin-resistant. Numerous studies have demonstrated that white coats worn by medical professionals are frequently contaminated with pathogenic bacteria, including both methicillin-resistant *S. aureus* (MRSA) and other pathogens [12, 20, 21]. Banu et al. [22] isolated *S. aureus* (methicillin-susceptible), MRSA, and *P. aeruginosa* from white coats of medical students and interns, Qaday et al. [7] isolated *S. aureus*, *E. coli*, and *P. aeruginosa* from white coats of physicians and medical students at a referral and teaching hospital, and Loh et al. [23] and Munoz-Price et al. [17] reported *S. aureus* and *Acinetobacter* spp. from white coats of physicians and medical students. Similarly, Gupta et al. [12] documented various pathogenic bacterial isolates including *Staphylococci*, *Streptococci*, *Salmonella*, *Pseudomonas* sp., vancomycin-resistant enterococci (VRE), *Klebsiella* sp., and *E. coli* from white coats of nurses at a tertiary care hospital. Although nonpathogenic bacterial contaminants have less clinical significance in the healthcare settings, these bacterial isolates have been reported by many authors from white coats of medical professionals [2, 17, 22]. The presence of potentially pathogenic bacteria on white coats may maximize the risk of transmission of infection in the healthcare setting.

## 5. Conclusions

This study showed a high degree of bacterial contamination on the nurses' white coats along with the presence of pathogens belonging to the WHO list of critical priority and high priority, thus posing a high risk of transmission to patients. There was a high burden of bacterial contamination on nurses' white coats which was more in second workdays as compared to first workdays. Therefore, proper maintenance and handling practices of the white coats should be undertaken to minimize the degree of bacterial contamination and to prevent cross-contamination of healthcare-associated pathogens in the hospital setting.

Freshly washed white coats should be used by nurses for every work shifts, and suitable strategies for decontamination of white coats should be implemented by the hospitals. Efforts should be made to discourage the usage of white coats outside clinical areas. We recommend further studies to compare the presence of bacteria across different hospital wards and other types of medical professionals and to see the seasonal trend of microorganisms. It is also recommended to do similar studies at facilities that wash coats for staff and require them to not wear outside the working area. Moreover, studies should be targeted to see if the isolates from white coats are similar to patient's clinical isolates during a defined period.

### 5.1. Limitations

The main limitation of the study was a smaller number of participants enrolled from selected departments to evaluate the bacterial contamination of their white coats. The result can vary depending upon the working practice of the participants. The study was conducted at a point of time, so this may not represent the true burden of contamination by pathogens year-round. The Hawthorne effect might have also influenced the result in the study as the participants were aware that they were in the study and had fabric attached to their coats.

## Figures and Tables

**Table 1:** Ward-wise distribution of bacterial load on fabric swatches after the first and second work shifts.

Wards	Work shift	Bacterial count (CFU/inch^2^)			
		Sample from nurses		Average count = (1 + 2)/2	Increase after second shift (%)
		1	2		
Medicine	I	125	94	109.5	54.3
	II	175	163	169	

Surgery	I	119	88	103.5	51.2
	II	206	107	156.5	

Postoperative	I	381	681	531	27.3
	II	475	877	676	

Maternity	I	1875	1125	1500	29.5
	II	2348	1537	1942.5	

Intensive care unit	I	500	487	493.5	248.2
	II	1875	1562	1718.5	

Pediatric	I	325	487	406	18.5
	II	394	568	481	

Total count in all six wards	I	3325	2962	3143.5	63.6
	II	5473	4814	5143.5	

Average count in each ward	I	554	494	524	63.6
	II	912	802	857	

Controls	No growth				

**Table 2:** Contamination of white coats with pathogens and nonpathogens.

Wards	Work shift	Pathogens				Opportunistic pathogens	Nonpathogens	
		A	B	C	D	E	F	G
Medicine	**I**	−	+	−	−	+	+	+
	II	−	−	−	−	+	+	+

Surgery	I	−	−	−	−	+	+	+
	II	—	−	−	−	+	+	+

Postoperative	I	−	−	+	−	+	+	+
	II	−	−	−	−	+	+	+

Maternity	I	−	−	−	−	+	+	+
	II	+	−	−	−	+	+	+

Intensive care	I	+	−	−	−	+	+	+
	II	−	−	−	−	+	+	+

Pediatric	I	−	−	−	+	+	+	+
	II	−	—	—	+	+	+	+

A = *Staphylococcus aureus*; B = *Escherichia coli*; C = *Pseudomonas aeruginosa*; D = *Acinetobacter* species; E = coagulase-negative staphylococci (CoNS); F = *Micrococcus* species; G = *Bacillus* species.

**Table 3:** Perception of nurses (*N* = 12) towards the handling of white coats.

Questions	Answer in percentage
How frequently do you wash your white coats?	
Three days or less	55.4
One week	44.4
Two to four weeks	0
One month or more	0

Where/how do you wash your coats?	
Laundering at hospital	0
Home wash	100

Where do you use your white coats?	
Only hospital	66.7
Hospital and outside (library, canteen, and garden)	33.3

How many white coats do you have?	
One	0
Two	62.5
More than two	37.5

Do you perceive a white coat to be clean if its collar and pocket are clean?	
Yes	44.4
No	55.6

## Data Availability

All data generated or analyzed during this study are included in this article.

## Abbreviations

ASM: American Society for Microbiology
CFU: Colony-forming unit
CHF: Congestive heart failure
CKD: Chronic kidney disease
CoNS: Coagulase-negative staphylococci
COPD: Chronic obstructive pulmonary disease
DM: Diabetes mellitus
HAI: Healthcare-associated infection
HCW: Healthcare worker
HIV: Human immunodeficiency virus
ICU: Intensive care unit
MRSA: Methicillin-resistant *Staphylococcus aureus*
SDH: Subdural hematoma
s/p: Status post
TB: Tuberculosis
UTI: Urinary tract infection
VRE: Vancomycin-resistant enterococci
WHO: World Health Organization.
